# Editorial: Competencies in veterinary education

**DOI:** 10.3389/fvets.2022.1007049

**Published:** 2022-09-14

**Authors:** Jared A. Danielson

**Affiliations:** Department of Veterinary Pathology, College of Veterinary Medicine, Iowa State University, Ames, IA, United States

**Keywords:** competency based veterinary education (CBVE), outcomes-based education, programmatic assessment, veterinary simulators, day 1 competencies, health informatics, employability, professional identity

Competency-based approaches to education are gaining popularity in the medical sciences. Recent development of the Competency Based Veterinary Education (CBVE) framework (1), entrustable professional activities (EPAs) (2), and milestones (3), as well as the growing number of presentations and publications regarding CBVE, demonstrate the rapid growth of interest in CBVE among veterinary educators. Competency based education (CBE) emphasizes (a) clear articulation of outcomes, (b) a logical progressive sequence of competencies, (c) competency-based, rather than time-based progression, (d) learning experiences tailored to the individual, and (e) an assessment approach based on numerous low-stakes, feedback-rich assessments carefully integrated into the educational program (4–6).

The growing popularity of CBVE invites consideration of a variety of important questions. For example, if veterinary licensure necessitates day-1 competence, what exactly must new graduates be competent to do? Can competence be universally defined, or is it tied to region or patient species? How is competence best measured? Is a competency-based approach better aligned with some curricular practices than others? What characteristics of veterinary school applicants are most predictive of subsequent competence? Perhaps most fundamental of all, does a competency-based educational approach actually produce more competent graduates? Answers to such broad questions could fill many manuscripts. This Research Topic does not focus exclusively any one of the foregoing questions, but contributes several pieces to the broader puzzle, both providing useful insight, as well as underscoring the breadth and complexity of answering such questions.

The contributions from Rienhard et al., Cake et al., and Ouyan et al. all provide a valuable perspective regarding historically under-emphasized outcomes in veterinary education. Reinhard et al.'s study underscores the importance of professional skills, with an emphasis on well-being and professional success, as well as professional identify development. Similarly, Cake et al. explore preparation for veterinary practice through the lens of employability as defined through the VetSet2Go Project. In their framework, veterinary capabilities comprise only one of five important overlapping domains; the other four include effective relationships, professional commitment, psychological resources, and self-awareness, and identity formation. Finally, Ouyang et al. explore the importance of new veterinary graduates' ability to assess, select, and implement the technologies associated with optimizing and providing healthcare, as well as promoting the work-life balance of the veterinary team. An important message of these three contributions is that traditional competence, defined primarily in terms of the practice of clinical veterinary medicine, is essential, but insufficient, to the preparation of day-1 ready veterinarians. This is particularly true if competence embraces all characteristics and abilities necessary for new graduates to thrive as veterinary professionals.

Several contributions focus on the importance of one or more aspects of assessment as it relates to competency-based education. Bok et al. remind us that there is far more to a competency-based approach than either identifying competencies, or designing and delivering curricula with competence in mind. Fundamental to competency-based education is an assessment approach that supports the development of competence. Programmatic assessment, with its use of competence committees, offers a powerful tool for aligning assessment practice with a competency-based focus. Tegzes and Frost similarly emphasize the role of assessment in competency-based veterinary education, focusing on the challenge (and opportunity) associated with assessing professionalism when working with members of other health professions. The Interprofessional Professional Assessment (IPA) is offered as an instrument for measuring behaviors in six competency domains associated with inter-professional proficiency: communication, respect, altruism and caring, excellence, ethics, and accountability.

Any educational approach, but especially one that is based on verifiable competence, requires the provision of high-quality opportunities for students to practice what they are learning. Two of this topic's articles emphasize such practice opportunities. Sheats et al.'s description of developing and evaluating a low-fidelity equine castration model illustrates that simple models for practicing clinical skills can provide students with meaningful practice prior to engaging in the targeted skill. Nessler et al. illustrate the principle of meaningful practice with a cognitive task—solving common veterinary neurology cases. In their study, prompted by an effort to engage students actively during the COVID-19 pandemic, students solved neurology cases presented electronically, with or without the addition of an outdoor scavenger hunt in which they explored their campus and the surrounding forest locating pre-determined GPS coordinates. Not only did students enjoy the outdoor scavenger hunt, but compared to students in the control group, students participating in the scavenger hunt scored higher on MCQ and free text questions about what they were learning. While neither of these studies are inherently or exclusively “competency-based,” they illustrate meaningful ways to engage students in beneficial practice—an important element of a competency-based educational approach.

Finally, while many find competency-based education compelling, adopting CBVE frequently demands resources, time, and hard work. Before embarking on such a challenging endeavor, colleges are wise to explore potential costs and benefits. Danielson's “Key Assumptions...” is intended to assist colleges in this exploration, describing six key assumptions underlying a competency-based approach to medical sciences education. Danielson briefly analyzes each assumption in terms of how well it is supported by the educational literature, as well as its goodness-of-fit to the context of veterinary medical education. While not all assumptions of CBME/CBVE are proven in the medical sciences education literature, available evidence suggests that a competency-based approach is promising for improving student learning, and for better preparing new graduates to take full advantage of the many opportunities available to veterinary professionals.

## Author contributions

The author confirms being the sole contributor of this work and has approved it for publication.

## Conflict of interest

The author declares that the research was conducted in the absence of any commercial or financial relationships that could be construed as a potential conflict of interest.

## Publisher's note

All claims expressed in this article are solely those of the authors and do not necessarily represent those of their affiliated organizations, or those of the publisher, the editors and the reviewers. Any product that may be evaluated in this article, or claim that may be made by its manufacturer, is not guaranteed or endorsed by the publisher.
